# Impact of COVID-19 on breast cancer care - a retrospective cohort study in Brazil

**DOI:** 10.1186/s12885-026-15994-4

**Published:** 2026-04-16

**Authors:** Diego Wallace Nascimento, José Roberto Filassi, Ângela Francisca Trinconi Cunha, Rodrigo Gonçalves, Marina Bellati Kuller, Jonathan Yugo Maesaka, Gabriela Boufelli de Freitas, Bruno Salvador Sobreira Lima, Yedda Nunes Reis, Rosa Maria Salani Mota, Rafael Pegado de Abreu Freitas, José Roberto Morales Piato, Luciana Rodrigues Carvalho Barros, Lorine Arias Bonifácio Teixeira, Silvia Radwanski Stuart, Maria Carolina Formigoni, Edmund Chada Baracat, José Maria Soares Júnior, Bruna Salani Mota

**Affiliations:** 1 Present Address: Department of Mastology, Division of Gynecology and Obstetrics, Faculty of Medicine of University of São Paulo, University of São Paulo, Arnaldo Avenue, 455, Sao Paulo, Sao Paulo Brazil; 2 Instituto do Câncer do Estado de São Paulo – Octavio Frias de Oliveira, University of São Paulo, Arnaldo Avenue, 251, São Paulo São Paulo, Brazil; 3 Division of Gynecology and Obstetrics, Faculty of Medicine of University of São Paulo, University of São Paulo, Arnaldo Avenue, 455, São Paulo, Brazil; 4 Federal University of Ceará, Fortaleza, Brazil; 5 Department of Radiotherapy, Faculty of Medicine University of São Paulo, São Paulo, Brazil; 6 Albert Einstein Israeli Hospital, São Paulo, Brazil

**Keywords:** Breast cancer, COVID-19 pandemic, Delivery of health care, Healthcare system

## Abstract

**Background:**

The COVID-19 pandemic disrupted healthcare systems worldwide and substantially affected breast cancer care. International reports have described delays in diagnosis, stage, and modifications in treatment strategies, however, little is known about how the pandemic has affected the entire sequence of steps from diagnosis to treatment initiation and completion within the Brazilian public healthcare system.

**Methods:**

This retrospective cohort study followed the STROBE guidelines and was conducted at a high-volume public cancer center in São Paulo, Brazil. A total of 1,306 women diagnosed with invasive or in situ breast cancer were included: 755 patients in the prepandemic period (March 2018–February 2019) and 551 patients in the pandemic period (March 2020–February 2021). Clinical, pathological, treatment, and time-related variables along the care pathway were extracted from electronic medical records and compared using appropriate statistical tests, with significance set at *p* < 0.05.

**Results:**

A 32% reduction in the number of treated patients occurred during the pandemic (551 vs. 755). Patients who were diagnosed during the pandemic were younger (48.3 vs. 53.6 years; *p* < 0.001) and presented with more advanced clinical stages, including higher rates of cT3–T4 tumors and nodal involvement (*p* < 0.001). The use of neoadjuvant therapy increased from 36.8% to 49%, particularly among early-stage and luminal B tumors. Key intervals across the care pathway were significantly prolonged, including time to initial treatment and completion of adjuvant therapy. Radiotherapy practices have shifted toward hypofractionated protocols.

**Conclusion:**

The first year of the COVID-19 pandemic was associated with fewer treated patients, stage migration toward more advanced disease, greater reliance on neoadjuvant therapy, and prolonged time intervals along the care pathway from diagnosis to treatment initiation and completion. These findings highlight the need for resilient oncology systems, protected treatment pathways, and strengthened care coordination to mitigate the impact of future healthcare crises.

**Supplementary Information:**

The online version contains supplementary material available at 10.1186/s12885-026-15994-4.

## Background

The COVID-19 pandemic, declared by the World Health Organization (WHO) in March 2020, resulted in the reallocation of financial resources and the implementation of measures to limit viral transmission within health systems [1]. In breast cancer, several studies have reported reductions in screening, delays in diagnosis, and a shift toward more advanced disease at presentation, raising concerns about long-term survival and increased treatment-related costs. These effects were observed across different health systems, including those in low- and middle-income countries, where resource reallocation and social distancing measures had disproportionate consequences [2–6]. 

In Brazil, the public healthcare system has undergone extensive restructuring to accommodate the surge of COVID-19 cases [7]. Screening programs were interrupted, elective surgeries were postponed, and diagnostic pathways were modified [3]. Studies from Brazilian centers have already demonstrated substantial increases in late-stage breast cancer diagnoses during the pandemic. However, these investigations have focused primarily on-stage distribution or short-term procedural volumes rather than on the full continuum of breast cancer care [6].

A critical gap persists regarding how the pandemic affects the sequence of steps from diagnosis to treatment initiation and completion. Understanding disruptions along this care pathway is essential, particularly in high-complexity public cancer centers that serve as national referral hubs and already operate near capacity. The extent to which delays occur at each step—diagnostic confirmation, treatment initiation, and adjuvant treatment completion—remains poorly characterized.

Thus, identifying how the pandemic has altered each step of the breast cancer care pathway—from diagnosis to treatment completion—is essential to understanding the magnitude of its impact on care delivery. This study aims to evaluate the impact of the COVID-19 pandemic on breast cancer diagnosis, staging, and treatment strategies, as well as on time intervals across the care pathway, within a major Brazilian public oncology center. By comparing prepandemic and pandemic cohorts, we sought to identify where delays occurred and how care patterns were modified during this unprecedented healthcare crisis.

## Methods

### Study design and setting

This retrospective cohort study was conducted at the Instituto do Câncer do Estado de São Paulo (ICESP), a high-complexity public cancer center affiliated with the University of São Paulo and part of the Hospital das Clínicas complex (HCFMUSP), the largest hospital complex in Latin America. The study followed the STrengthening the Reporting of OBservational studies in Epidemiology (STROBE) guidelines for observational research [8].

We included women diagnosed with invasive or in situ breast cancer during two distinct periods: the prepandemic cohort (March 2018–February 2019; *n* = 755) and the pandemic cohort (March 2020–February 2021; *n* = 551). These periods were selected to represent routine pre-COVID-19 activity and the most critical year of the pandemic in the region.

During the COVID-19 pandemic, HCFMUSP served as a national referral center for severe COVID-19 cases, which required substantial reorganization of hospital resources. The ICESP surgical capacity was significantly reduced, with approximately 62.5% of operating rooms (10 of 16) converted to intensive care unit beds, directly impacting oncological care delivery.

Despite these constraints, ICESP maintained medical oncology, radiation oncology, and breast surgical oncology services throughout the study period. Institutional protocols were implemented to mitigate viral transmission, including systematic COVID-19 screening prior to elective surgical procedures, reorganization of outpatient and inpatient patient flow, and enhanced infection control measures. Treatment prioritization strategies were adopted based on disease stage and clinical urgency, and the use of neoadjuvant therapies was expanded when appropriate. While some healthcare professionals were temporarily reassigned to support COVID-19–related services, resolute oncology teams, including physicians and nursing staff, continued to provide cancer care.

ICESP is a high-complexity public oncology center within the Brazilian Unified Health System (SUS) and routinely receives referrals of complex cancer cases from across Brazil [9]. Its location in Southeast Brazil—the region with the highest incidence of breast cancer in the country [10]—underscores its strategic role and helps explain the sustained demand for oncological care observed during the pandemic period.

ICESP is formally classified as a Center of High Complexity in Oncology (Centro de Assistência de Alta Complexidade em Oncologia – CACON) within the Brazilian Unified Health System (SUS). CACONs comprise national reference institutions, such as the National Cancer Institute (INCA), and large tertiary referral hospitals that concentrate complex oncological care and generate national data on access to diagnosis and treatment. CACONs are tertiary-level public oncology centers accredited by the Brazilian Ministry of Health to provide comprehensive cancer care, including diagnostic evaluation, surgical oncology, systemic therapy, and radiotherapy, with integrated multidisciplinary teams. These centers function as regional or national referral hubs for complex cancer cases and are required to meet structural, technological, and staffing standards defined by national oncology care policies [11]. 

### Eligibility criteria

We included all female patients who were diagnosed with invasive breast cancer or ductal carcinoma in situ during two predefined periods and who completed all cancer-directed treatment at the ICESP. We excluded patients who were diagnosed with *de novo* stage IV disease outside the defined periods, those whose treatment was deferred due to severe comorbidities, individuals with synchronous malignancies, and those who discontinued or transferred care before completing treatment.

### Data collection and variables

Data were extracted from electronic medical records and entered Research Electronic Data Capture (*REDCap*) by trained breast surgeons [12]. The variables included patient demographics, tumor characteristics (histology, molecular subtype, and clinical TNM staging), diagnostic timelines, and treatment modalities (surgery, systemic therapy, radiotherapy). The molecular classification was defined as follows: estrogen and progesterone receptors were considered positive in ≥ 1% of the stained cells. Luminal A and B tumors were distinguished by a Ki-67 cutoff index of 14%, HER2 positivity was defined as an immunohistochemistry score of 3 + or positive by in situ hybridization (for 2 + scores). The first appointment was defined as the patient’s initial breast cancer evaluation at the ICESP. The time intervals assessed included biopsy-to-admission, first appointment-to-initial treatment, and completion times for surgery, chemotherapy, and radiotherapy.

### Statistical analysis

Continuous variables are reported as the means ± standard deviations or medians with interquartile ranges, according to the distribution assessed by the Shapiro–Wilk test. Comparisons between cohorts were performed via Student’s *t* test or the Mann–Whitney U test for continuous variables and via Pearson’s chi-square test or Fisher’s exact test for categorical variables. Significance was established at *p* < 0.05. Analyses were conducted via IBM SPSS Statistics and Wizard Pro.

### Objectives

#### Primary objectives

To evaluate the impact of the COVID-19 pandemic on breast cancer management in a high-complexity public oncology center (CACON) within the Brazilian Unified Health System by comparing initial treatment strategies and time intervals across the care pathway between the prepandemic and pandemic periods.

To assess the impact of the pandemic on key care pathway intervals, including time from biopsy to admission, time from first appointment to treatment initiation, and time to completion of surgery, adjuvant chemotherapy, and radiotherapy.

#### Secondary objectives

To compare demographic and tumor characteristics (age, molecular subtype, histology, and clinical cT and cN stages) between patients diagnosed before and during the pandemic. Changes in therapeutic patterns, including the use of neoadjuvant treatment, types of surgery and frequency of immediate breast reconstruction, and modifications in radiotherapy protocols, particularly the adoption of hypofractionated regimens, should be assessed. To quantify the magnitude of the reduction in the number of cases treated and explore its potential determinants within the institutional context.

### Ethics approval and consent to participate

The study was approved by the Ethics Committee of the Hospital das Clínicas, Faculdade de Medicina da Universidade de São Paulo (HCFMUSP), São Paulo, Brazil (CAAE: 52378521.8.0000.0068).

All procedures were performed in accordance with the ethical standards of the institutional and national research committee and with the 1964 Declaration of Helsinki and its later amendments.

Given the retrospective design of the study and the use of anonymized data, the requirement for informed consent was waived by the Ethics Committee. Strict confidentiality of medical records was maintained throughout the study.

## Results

### Study population

#### Clinical characteristics of the participants

**Fig. 1:**
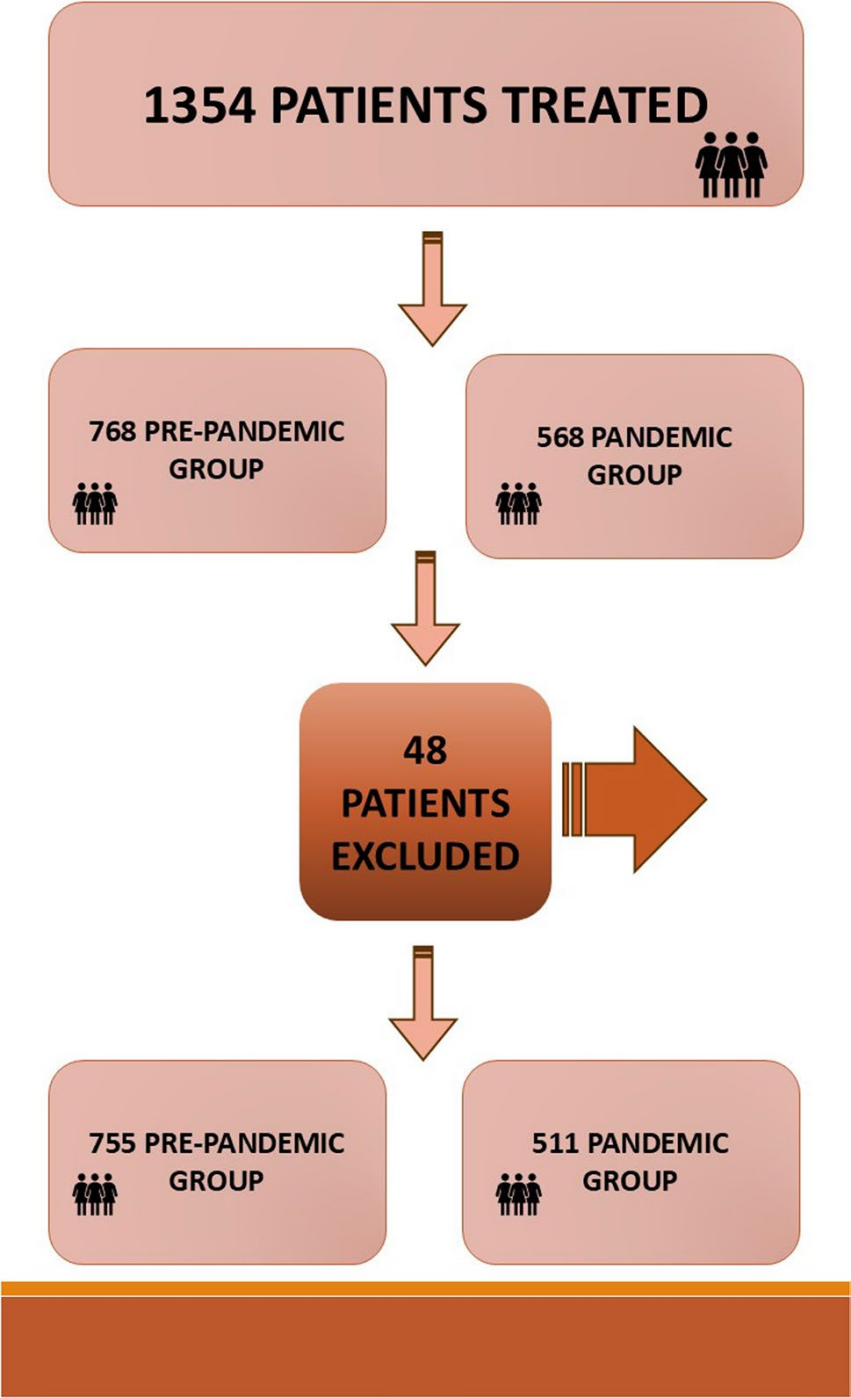
Total number of prepandemic and pandemic patients

**Fig. 2:**
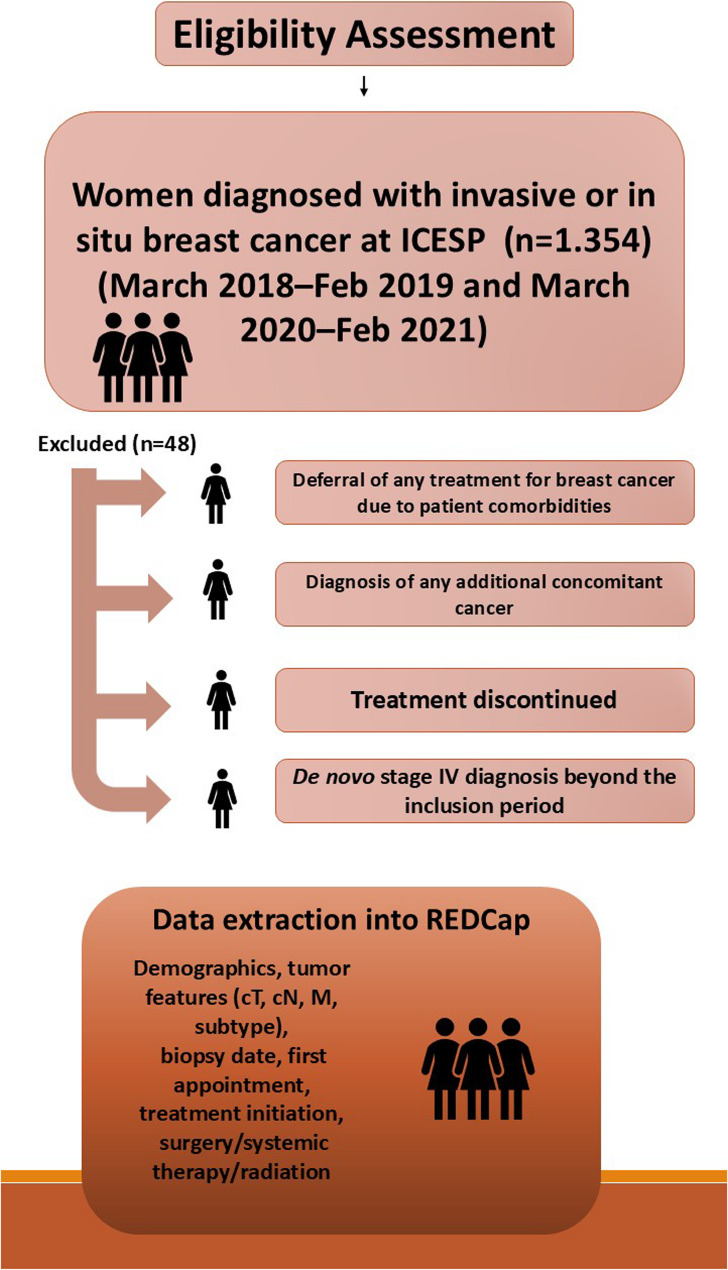
Eligibility assessment

Of the 1,354 patients initially screened, 1,306 met the eligibility criteria and were included in the analytical cohort: 755 in the prepandemic period and 551 during the pandemic. A total of forty-eight individuals (3.5%) were excluded due to de novo stage IV disease identified outside the study window, competing comorbidities resulting in treatment deferral, synchronous malignancies, or incomplete treatment pathways. This corresponded to a 32% reduction in the number of patients receiving breast cancer care during the pandemic year (Figs. 1 and 2).

#### Clinical characteristics of the participants

Patients diagnosed during the pandemic were significantly younger, with a mean of 48.3 (23.5–92.2) years versus 53.6 (22.1–98.2) years, *p* < 0.001. BMI, histologic subtype, and molecular classification distributions were similar between the cohorts.

Clinical staging at presentation demonstrated a shift toward more advanced disease in the pandemic cohort. The cT-stage distribution was as follows: cT1 (pandemic: 15.1%, prepandemic: 17.7%), cT2 (pandemic: 32.0%, prepandemic: 31.7%), cT3 (pandemic: 27.2%, prepandemic: 21.9%), and cT4 (pandemic: 22.4%, prepandemic: 19.9%), *p* < 0.001. Overall, the proportion of patients presenting with cT2–cT4 tumors increased during the pandemic period. The cN stage distributions were as follows: cN0 (pandemic: 48.4%, prepandemic: 55.1%), cN1 (pandemic: 37.9%, prepandemic: 30.8%), cN2 (pandemic: 10.9%, prepandemic: 12.0%), and cN3 (pandemic: 2.8%, prepandemic: 2.0%), *p* < 0.001. These findings further support the pattern of more advanced clinical presentation during the pandemic (Table 1).

**Table 1:** Clinical characteristics, surgical strategies, reconstruction modalities, and chemotherapy and radiotherapy regimens

	Pre-pandemic Group	Pandemic Group	*p*
**Age**	Mean( Range)	Mean( Range)	< 0.001
	53.62 ( 22.1–98.2)	48.28 ( 23.5–92.2)	
**BMI**			0.14
	28.41( 16 -55.5)	28.89( 16.6–51.3)	
**Clinical stage of breast - cT**	n(%)	n(%)	< 0.001
Tis	67(8.9)	17(3.3)	
T1	134(17.7)	77(15.1)	
T2	239(31.7)	164(32.0)	
T3	165(21.9)	139(27.2)	
T4	150(19.9)	114(22.4)	
**Clinical stage axilla - cN**			0.035
N0	416 (55.1)	247(48.4)	
N1	233(30.8)	194(37.9)	
N2	91(12.0)	56(10.9)	
N3	15(2.0)	14(2.8)	
TNM			< 0.001
0	64(8.5)	20(3.9)	
I	214(28.3)	141(27.5)	
II	180(23.9)	134(26.2)	
III	197(26)	149(29.1)	
IV	100(13.3)	68(13.4)	
Molecular classification			0.270
Luminal A	88(11.7)	45(8.9)	
Luminal B	372(49.2)	247(48.4)	
HER2	59(7.8)	42(8.3)	
TNBC	134(17.8)	86(16.8)	
Luminal HER2	103(13.6)	90(17.6)	
**Histological subtype**			0.806
IDC	596(78.9)	406(79.4)	
ILC	42(5.6%)	30(5.9%)	
DCIS	42 (5.6%)	22(4.3%)	
Others	76(10.0%)	53(10.4%)	
**Surgery Performed**			0.32
Breast Conserving Surgery	399(52.9)	262(51.3)	
Mastectomy	356(47.1)	249(48.7)	
**Breast Reconstruction**			0.66
No	464(61.5)	308(60.2)	
Yes	291(38.5)	203(39.8)	
**Reconstruction techniques/ Associations**			-
Flaps	212(28.1)	112(22.0)	
Prosthesis	331(43.8)	247(48.4)	
Flaps and prosthesis	13(1.7)	19(3.8)	
Oncoplastic (mammoplasty)	159(21.1)	109(21.4)	
Flaps and oncoplastic	13(1.7)	3(0.5)	
Prosthesis and oncoplastic	25(3.3)	19(3.8)	
Flaps, prosthesis and oncoplastic	3(0.4)	0(0.0)	
**Chemotherapy**			0.11
AC-T or ACTH	570(75.5)	423(82.7)	
TC or TCH	97(12.8)	40(7.8)	
T or TH	28(3.7)	14(2.8)	
FEC	2(0.2)	0(0.00)	
Others	48(6.3)	30(5.9)	
No information	11(1.4)	4(0.70)	
**RT Gy Dose**			< 0.001
26 Gy in 5 fractions	13(1.7)	24(4.7)	
40.05 Gy in 15 fractions	500(66.2)	355(69.4)	
48.01 Gy in 18 fractions	112(14.8)	112(21.9)	
Others	131(17.3)	20(4)	

*Abbreviations*: *AC-T *Doxorubicin, Cyclophosphamide, Paclitaxel, and Trastuzumab, *BMI *Body Mass Index, *DCIS *Ductal Carcinoma in Situ, *FEC *5-fluorouracil, Epirubicin, and Cyclophosphamide, *Gy *Gray, *IDC *Invasive Ductal Carcinoma, *ILC *Invasive Lobular Carcinoma, *RT *Radiotherapy, *TC * Docetaxel and Cyclophosphamide, *TNBC *Triple Negative Breast Cancer, *TCH *Docetaxel, Cyclophosphamide, and Herceptin

#### Initial treatment proposed

The initial breast cancer treatment differed between the pandemic group and the prepandemic group. Neoadjuvant therapy was more frequently used during the pandemic (49% vs. 36.8% before the pandemic). Upfront surgery decreased from 50.3% to 38.1%, whereas rates of palliative treatment remained similar (Table 2).

**Table 2:** Proposed initial treatment

Proposed Initial Treatment	Pre-pandemic Group -*n*(%)	Pandemic Group -*n*(%)	*p*	OR	95% CI
**Neoadjuvant treatment**	278(36.8%)	250(49.0%)	< 0.001	1.717	(1.352–2.157)
**Surgery**	380(50.3%)	195(38.1%)	-	1.000	(0.930 − 1.839)
**Palliative treatment**	97(12.8%)	66(12.9%)	0.13	1.304	(0.930 − 1.839)

*Abbreviations*: *OD *Odds Ratio

In the subgroup analysis, an increase in the indications for neoadjuvant treatment for stages I, II, and III disease was observed during the pandemic, with distributions of 19.9%, 62.9%, and 94.3%, respectively, compared with 12.4%, 50%, and 86.2%, respectively (Table 3).

**Table 3:** Initial treatment proposed according to staging and molecular classification

	Proposed Initial Treatment	Pre-pandemic Group -*n*(%)	Pandemic Group -*n*(%)	*p*
**TNM**				
0	Neoadjuvant	2(3.1%)	0(0.0%)	-
	Surgery	62(96.9%)	20(100.0%)	
I	Neoadjuvant	27(12.4%)	28(19.9%)	0.067
	Surgery	187(87.6%)	113(80.1%)	
II	Neoadjuvant	60(50.0%)	84(62.9%)	0.028
	Surgery	60(50.0%)	50(37.1%)	
III	Neoadjuvant	170(86.2%)	141(94.3%)	0.208
	Surgery	27(13.8%)	8(5.7%)	
IV	Systemic	64(63.6%)	47(68.8%)	1
	Surgery	36(36.4%)	21(31.3%)	
**Molecular classification**				
Luminal A	Neoadjuvant	11(12.2%)	8(17.1%)	0.575
	Surgery	77(87.8%)	37(82.9%)	
Luminal B	Neoadjuvant	133(35.7%)	126(51.0%)	0.001
	Surgery	239(64.3%)	121(49.0%)	
HER2	Neoadjuvant	31(52.4%)	28(67.6%)	0.251
	Surgery	28(47.6%)	14(32.4%)	
TNBC	Neoadjuvant	107(80.2%)	71(82.4%)	0.847
	Surgery	27(19.8%)	15(17.6%)	
Luminal-HER2	Neoadjuvant	50(48.1%)	57(63.5%)	0.072
	Surgery	53(51.9%)	33(36.5%)	

*Abbreviations*: *TNBC *Triple Negative Breast Cancer

In terms of molecular classification, there was also an important increase in the indication for neoadjuvant treatment among patients with a luminal B molecular profile during the pandemic, with 51% of patients starting with neoadjuvant treatment, compared with 35.7% in the prepandemic period. Luminal-HER2 patients were more likely to receive neoadjuvant treatment during the pandemic (63.5% vs. 48.1% in the prepandemic group), but this difference was not statistically significant, as shown in Table 3.

#### Surgical procedures

The distribution of oncologic surgical procedures did not differ significantly between cohorts. Immediate breast reconstruction was performed in 39.8% surgical cases during the pandemic and 38.5% before the pandemic. A greater proportion of prosthesis-based reconstruction was observed during the pandemic (48.4% vs. 43.8%), whereas the proportion of flap-based techniques slightly decreased (Table 1).

#### Radiotherapy

Radiotherapy demonstrated a substantial change in favor of hypofractionated regimens. The use of 26 Gy in 5 fractions increased from 1.7% to 4.7%, and the use of 40.05 Gy in 15 fractions increased from 66.2% to 69.4% (*p* < 0.001). The use of longer regimen declined proportionally (Table 1).

#### Care pathway intervals and treatment timelines

Most intervals across the breast cancer care pathway were prolonged during the pandemic, although the magnitude of delay varied according to the specific step. Statistically significant delays were observed in the time from biopsy to first appointment, from first appointment to treatment initiation, and in the time to completion of additional adjuvant treatment, as described below.

#### Time from biopsy to admission

The median time from breast cancer diagnostic biopsy to the first appointment decreased during the pandemic, from 40 days (IQR 27–55) in the prepandemic period to 36 days (IQR 26–47) during the pandemic (*p* < 0.001).

#### Time from first appointment to initial treatment

The median time to neoadjuvant chemotherapy as the initial treatment was 29 days (IQR 16–49.75) during the pandemic, whereas it was 24 days (IQR 15–41.25) before the pandemic (*p* = 0.04). The median time to undergo surgery as the first treatment was 77 days (IQR 51–110) during the pandemic, in contrast to 51 days (IQR 38–71) before the pandemic (*p* < 0.001).

#### Time to complete additional adjuvant treatment

The median time to surgery after neoadjuvant treatment differed across periods; during the pandemic, it was 224 days (IQR 199–247.5), rather than 210 days (IQR 191–232) before the pandemic (*p* = 0.007). The median time frame for adjuvant chemotherapy was 127 days (IQR 105–163) during the pandemic, whereas it was 108 days (IQR 85.5–368) prior to the pandemic (*p* = 0.001). The median time to complete adjuvant radiotherapy was 282 days (IQR 72–763) during the pandemic versus 259 days (IQR 14–1031) before the pandemic (*p* < 0.001) (Table 4).

**Table 4:** Patients' time in the hospital (in days)

	Pre-pandemic Group	Pandemic Group	*p*
**Time in days from biopsy to admission to the service**			
	40 (IQR 27–55)	36 (IQR 26–47)	*p* < 0.001
**Time in days from the first consultation at the ICESP breast surgery service to the start of the first treatment - first neoadjuvant treatment**			
	24 (IQR 15–41.25)	29 (IQR 16–49.75)	0.04
**Time in days from the first consultation at the ICESP breast surgery service to the start of the first treatment - first upfront surgery treatment**			
	51 (38–71)	77 (51–110)	*p* < 0.001
**Time in days to complete additional treatments, considering the date of the first institutional consultation - Surgery after neoadjuvant treatment**			
	210 (IQR 191–232)	224 (IQR 199–247.5)	0.007
**Time in days to complete additional treatments, considering the date of the first institutional consultation - Adjuvant chemotherapy, post-upfront surgery**			
	108 (IQR 85.5–368)	127 (IQR 105–163)	0.001
**Time in days to complete adjuvant radiotherapy, considering the date of diagnosis**			
	259 (IQR 14–1,031)	282(IQR 72–763)	*p* < 0.001

## Discussion

This study provides a comprehensive evaluation of the impact of the COVID-19 pandemic on breast cancer care in a Brazilian public oncology center. During the first year of the pandemic, there was a 32% reduction in the number of treated cases, accompanied by stage migration toward more advanced disease (stages III and IV) and a reduction in diagnoses of ductal carcinoma in situ. Similar patterns have been reported nationally and internationally. In Brazil, Rocha et al. observed a 10.7% increase in stage III/IV breast cancer diagnoses between 2020 and 2021 compared with 2013–2019 [2], while Bonadio et al. reported that 37.3% of patients diagnosed between September 2020 and January 2021 presented with stage III disease, compared with 23.2% in the previous year [6]. Consistent findings were also reported in a multicenter study from Japan, in which the proportion of patients diagnosed with stage IIB or higher disease increased from 22.0% to 31.3% during the pandemic period [13]. Together, these data indicate that pandemic-related disruptions in cancer detection represent a global phenomenon rather than an isolated effect in low- or middle-income settings.

In parallel with stage migration, important changes in treatment strategies were observed. In our cohort, increased use of neoadjuvant therapies and prolonged time intervals along the care pathway from diagnosis to treatment initiation were noted, particularly with respect to the interval between biopsy and the start of therapy. These findings align with reports from other settings, in which neoadjuvant approaches were more frequently adopted during the pandemic. Habbous et al. reported a two- to threefold increase in the use of neoadjuvant therapies compared with the prepandemic period [14], and Khorfan et al. observed that patients with stage I luminal tumors were three times more likely to receive neoadjuvant endocrine therapy during the pandemic [15]. Such adaptations reflect efforts to mitigate surgical delays and optimize disease control amid constrained healthcare resources.

### Adaptations in breast cancer treatment delivery during the COVID-19 pandemic

In response to pandemic-related constraints, substantial adaptations in breast cancer treatment delivery were implemented. Consistent with international experience [15], our cohort showed a marked increase in neoadjuvant treatment utilization, from 36.8% in the prepandemic period to 49% during the pandemic. This strategy enabled postponement of surgical procedures while maintaining disease control, which was particularly relevant in the context of a 62.5% reduction in operating room capacity at our institution.

The impact of the pandemic on surgical volume has been documented across different healthcare systems. Boyd et al. reported a 6.9% reduction in oncological surgeries in New York [16], while Ribeiro et al. observed a 15.7% decrease in oncological procedures in Brazil during 2020 [3]. Breast reconstruction was also disproportionately affected, with a reported 42.9% reduction in immediate autologous tissue reconstruction [16, 17]. In our institution, a more pronounced reduction in surgical volume was observed, with a 32.4% decrease in treated cases, from 755 patients in the prepandemic period to 511 during the pandemic. Despite this reduction, the distribution of breast surgical procedures and reconstructive approaches remained similar across periods, reflecting institutional efforts to preserve standard surgical practices whenever feasible.

Radiotherapy protocols were likewise adapted in accordance with international recommendations aimed at minimizing hospital visits and reducing exposure risk. The increased adoption of hypofractionated regimens during the pandemic, including 26 Gy in five fractions (from 1.7% to 4.7%) and 40.05 Gy in 15 fractions (from 66.2% to 69.4%), illustrates how radiotherapy services adjusted to pandemic constraints while maintaining treatment efficacy. These adaptations are supported by robust evidence demonstrating the safety and effectiveness of hypofractionated radiotherapy schedules in breast cancer management [18, 19].

### Molecular subtype distribution and treatment implications

A noteworthy finding in our cohort was the higher prevalence of the luminal B molecular subtype compared with luminal A, an inverse pattern to what is commonly described in unselected breast cancer populations [20]. This finding may be explained by the referral profile of our institution as a high-complexity tertiary center, potentially leading to referral bias toward biologically more aggressive tumors, such as luminal B disease [21]. This distribution is consistent with the observed therapeutic strategies, as the higher prevalence of luminal B tumors contributed to the increased use of neoadjuvant endocrine therapy during the pandemic. Although luminal B tumors are frequently managed with upfront surgery under routine conditions, treatment decisions during the pandemic were influenced by additional factors beyond molecular subtype alone, including more locally advanced primary tumors, greater nodal involvement at presentation, and the need to postpone surgical procedures due to limited operating room availability. In this context, neoadjuvant endocrine therapy may have been preferentially selected as a strategy to maintain disease control while deferring surgery to patients who might otherwise have been candidates for upfront surgical management.

### Multifactorial causes of treatment delays

The COVID-19 pandemic significantly prolonged time to treatment initiation across all therapeutic modalities. Neoadjuvant chemotherapy initiation was delayed by a median of five days, whereas upfront surgery experienced the most substantial delay, with a median increase of twenty-six days. Post-neoadjuvant surgery and adjuvant chemotherapy were postponed for fourteen and nineteen days, respectively. When interpreted within the context of the Brazilian “60-day Law” (Federal Law No. 12.732/2012) [22], which mandates initiation of cancer treatment within 60 days of pathological diagnosis in the public healthcare system, these findings illustrate how pandemic-related disruptions added strain to timelines that are already challenging to meet in routine clinical practice [10]. These delays resulted from multiple interconnected factors that accumulated along the care pathway, creating a cascade effect that impacted the entire treatment trajectory.

The preserved institutional performance observed during the pandemic, when compared with national pre-pandemic reports, should be interpreted within the local organizational context of ICESP. As a high-volume tertiary referral CACON located in Southeast Brazil, ICESP benefits from integrated diagnostic and treatment services, established multidisciplinary workflows, and prioritization strategies that were rapidly adapted during the COVID-19 crisis. These institutional characteristics may have mitigated, but not eliminated, pandemic-related delays. Importantly, such organizational capacity and resource availability are not uniformly distributed across the Brazilian Unified Health System (SUS) [23]. Therefore, our findings should not be interpreted as representative of the entire SUS network, but rather as an illustration of how structured organizational strategies within high-complexity centers may partially preserve cancer care delivery during health system disruptions.

The diagnostic pathway faced unprecedented challenges during the pandemic. Initial delays were related to the need to reassess external diagnostic examinations, complete incomplete workups, and ensure adequate systemic staging before treatment initiation. These challenges were compounded by reduced pathology laboratory capacity, as personnel and resources were partially redirected to support COVID-19 testing demands. In addition, the implementation of infection control measures introduced further logistical constraints. All patients scheduled for elective procedures underwent mandatory COVID-19 screening, including RT-PCR testing [24, 25] and, when clinically indicated, non-contrast chest computed tomography [18]. Patients presenting with respiratory symptoms experienced additional delays, as surgical procedures were postponed until complete clinical recovery and confirmation of a negative COVID-19 status.

Beyond structural and organizational factors, patient-level barriers also contributed to delays. Fear of hospital-acquired COVID-19 infection emerged as an important factor influencing delayed presentation and appointment cancellations. Mandatory quarantine measures and transportation restrictions disproportionately affected patients traveling from distant regions, further extending treatment timelines.

Although telemedicine emerged as an important strategy to maintain continuity of care during the pandemic, its rapid implementation required substantial adaptation by both healthcare providers and patients. During the initial phases, this transition often slowed, rather than expedited, care delivery. Concurrently, healthcare systems faced the challenge of balancing oncological care with fluctuating COVID-19 surges, necessitating continuous adjustments to surgical scheduling and resource allocation. This dynamic environment transformed previously routine processes into complex logistical challenges across all stages of cancer care.

## Strengths and limitations

This study is strengthened by its large sample size and comprehensive evaluation of time intervals across the breast cancer care pathway in a high-volume public oncology center. However, its retrospective design represents the primary limitation and may introduce selection and reporting biases. Patient exclusions were necessary to ensure internal validity by focusing on individuals who completed standard diagnostic and therapeutic pathways (Table 5). Given the small size and distinct clinical profile of the excluded group (*n* = 48, 3.5%), these exclusions are unlikely to compromise the external validity of the findings.

**Table 5:** Rationale for forty- eight exclusions

Exclusion Category	Rationale for Exclusion
De novo stage IV diagnosis beyond inclusion period	These patients follow distinct diagnostic and therapeutic pathways.
Deferral of treatment due to comorbidities	Delays were unrelated to systemic or pandemic factors, but individual clinical contraindications.
Additional concomitant malignancy	Multiple cancers alter treatment prioritization and timelines.
Withdrawal from treatment	Lack of completion of diagnostic or therapeutic steps along the care pathway prevents time-to-event assessments.

Data completeness during the pandemic may also have been affected by healthcare system overload and staff reallocation. Nevertheless, the use of electronic medical records and structured data systems, such as REDCap, helped mitigate reporting bias and allowed for a more accurate assessment of the pandemic’s impact on care delivery. Finally, as this study reflects the experience of a single public cancer center, caution is warranted when extrapolating the findings to other regions or healthcare settings with different organizational structures and resource availability.

## Conclusion

In the first year of the COVID-19 pandemic, ICESP managed approximately one-third fewer breast cancer cases, with a higher proportion of advanced-stage diagnoses and increased use of neoadjuvant therapies for stages I to III. In addition, the time required to complete oncological treatment increased significantly, reflecting prolonged intervals along the care pathway from the first appointment to completion of adjuvant therapy.

Together, these findings demonstrate how pandemic-related disruptions can accumulate across diagnostic and treatment stages, compromising timely cancer care in high-complexity public oncology centers. Long-term follow-up of this cohort will be essential to determine whether these delays and treatment adaptations translate into differences in survival and recurrence outcomes.

From a health system perspective, the experience of the COVID-19 pandemic highlights the need for resilient oncology services capable of maintaining essential cancer care during public health emergencies. Preserving protected oncological care pathways, implementing risk-stratified treatment protocols, ensuring diagnostic and surgical capacity, and strengthening care coordination mechanisms should be central components of future pandemic preparedness strategies.

## Supplementary Information

Supplementary Material 1.

## Data Availability

The raw data underlying this study are securely stored in the institutional REDCap database. These data may be made available to the journal’s reviewers upon formal request, in accordance with editorial policies. However, due to the Brazilian General Data Protection Law (Lei Geral de Proteção de Dados Pessoais – Federal Law No. 13,709/2018) and the presence of identifiable patient information (names and medical record numbers), the dataset cannot be made publicly accessible in open online repositories. Any data sharing must occur in a controlled manner and fully comply with applicable legal and ethical requirements.

## Abbreviations

BMI: Body Mass Index
CAAE: Certificado de Apresentação para Apreciação Ética
CACON: Center of High Complexity in Oncology
HCFMUSP: Hospital das Clínicas, Faculty of Medicine, University of São Paulo
ICESP: Instituto do Câncer do Estado de São Paulo
INCA: National Cancer Institute
REDCap: Research Electronic Data Capture
STROBE: Strengthening the Reporting of Observational Studies in Epidemiology
SUS: Unified Public Health System (Sistema Único de Saúde)
WHO: World Health Organization
