# Supplementary material for: Impact of COVID-19 on breast cancer care - a retrospective cohort study in Brazil
Source: BMC Cancer. 2026 Apr 16;26:675. doi: 10.1186/s12885-026-15994-4 (PMC13214390; doi:10.1186/s12885-026-15994-4)
Supplement: Supplementary file 1 — Supplementary Material 1. [file 12885_2026_15994_MOESM1_ESM.docx]

Title: Navigating the Crisis : Impact of COVID-19 on Breast Cancer Care - A Retrospective Cohort Study in Brazil STROBE Statement—checklist of items that should be included in reports of observational studies

|  | **Ite m No.** |  | **Page No.** | **Relevant text from manuscript** |
| --- | --- | --- | --- | --- |
|  |  | **Recommendation** |  |  |
| **Title and abstract** | 1 | (*a*) Indicate the study’s design with a commonly used term in the title or the abstract | 1 |  |
|  |  | (*b*) Provide in the abstract an informative and balanced summary of what was done and what was  found | 3 |  |
| **Introduction** |  |  |  |  |
| Background/rational  e | 2 | Explain the scientific background and rationale for the investigation being reported | 4 |  |
| Objectives | 3 | State specific objectives, including any prespecified hypotheses | 6 |  |
| **Methods** |  |  |  |  |
| Study design | 4 | Present key elements of study design early in the paper | 5 |  |
| Setting | 5 | Describe the setting, locations, and relevant dates, including periods of recruitment, exposure,  follow-up, and data collection | 5-6 |  |
| Participants | 6 | (*a*) *Cohort study*—Give the eligibility criteria, and the sources and methods of selection of  participants. Describe methods of follow-up | 5-6 |  |
|  |  | (*b*) *Cohort study*—For matched studies, give matching criteria and number of exposed and  unexposed | 5-6 |  |
| Variables | 7 | Clearly define all outcomes, exposures, predictors, potential confounders, and effect modifiers.  Give diagnostic criteria, if applicable | 5-6 |  |
| Data sources/  measurement | 8* | For each variable of interest, give sources of data and details of methods of assessment  (measurement). Describe comparability of assessment methods if there is more than one group | 5-6 |  |
| Bias | 9 | Describe any efforts to address potential sources of bias | 5-6; 15 |  |
| Study size | 10 | Explain how the study size was arrived at | 5-6 |  |

Continued on next page

| Quantitative  variables | 11 | Explain how quantitative variables were handled in the analyses. If applicable, describe which  groupings were chosen and why | 5-6 |
| --- | --- | --- | --- |
| Statistical  methods | 12 | (*a*) Describe all statistical methods, including those used to control for confounding | 5-6 |
|  |  | (*b*) Describe any methods used to examine subgroups and interactions | 5-6 |
|  |  | (*c*) Explain how missing data were addressed | 5-6 |
|  |  | (*d*) *Cohort study*—If applicable, explain how loss to follow-up was addressed | 5-6 |
|  |  | (*e*) Describe any sensitivity analyses | 5-6 |
| **Results** |  |  |  |
| Participants | 13* | (a) Report numbers of individuals at each stage of study—eg numbers potentially eligible, examined  for eligibility, confirmed eligible, included in the study, completing follow-up, and analysed | 7-11 |
|  |  | (b) Give reasons for non-participation at each stage | 7-11 |
|  |  | (c) Consider use of a flow diagram | 8 |
| Descriptive  data | 14* | (a) Give characteristics of study participants (eg demographic, clinical, social) and information on  exposures and potential confounders | 7-11 |
|  |  | (b) Indicate number of participants with missing data for each variable of interest | - |
|  |  | (c) *Cohort study*—Summarise follow-up time (eg, average and total amount) | 7-11 |
| Outcome data | 15* | *Cohort study*—Report numbers of outcome events or summary measures over time | *-* |
| Main results | 16 | (*a*) Give unadjusted estimates and, if applicable, confounder-adjusted estimates and their precision (eg, 95% confidence interval). Make clear which confounders were adjusted for and why they were  included | 7-11 |
|  |  | (*b*) Report category boundaries when continuous variables were categorized | 7-11 |
|  |  | (*c*) If relevant, consider translating estimates of relative risk into absolute risk for a meaningful time  period | 7-11 |

Continued on next page

| Other analyses | 1  7 | Report other analyses done—eg analyses of subgroups and interactions, and sensitivity analyses | - |
| --- | --- | --- | --- |
| **Discussion** |  |  |  |
| Key results | 1  8 | Summarise key results with reference to study objectives | 11-17 |
| Limitations | 1  9 | Discuss limitations of the study, taking into account sources of potential bias or imprecision. Discuss  both direction and magnitude of any potential bias | 11-17 |
| Interpretation | 2  0 | Give a cautious overall interpretation of results considering objectives, limitations, multiplicity of  analyses, results from similar studies, and other relevant evidence | 11-17 |
| Generalisabilit  y | 2  1 | Discuss the generalisability (external validity) of the study results | 11-17 |
| **Other information** | | | |
| Funding | 2  2 | Give the source of funding and the role of the funders for the present study and, if applicable, for the  original study on which the present article is based | 2 |
